# Supplementary material for: The causal relationship between the human gut microbiota and pyogenic arthritis: a Mendelian randomization study
Source: Front Cell Infect Microbiol. 2024 Nov 26;14:1452480. doi: 10.3389/fcimb.2024.1452480 (PMC11629706; doi:10.3389/fcimb.2024.1452480)
Supplement: Supplementary file 1 [file DataSheet1.zip › mendelian test/ebi-a-GCST90027525.csv_forest.pdf]

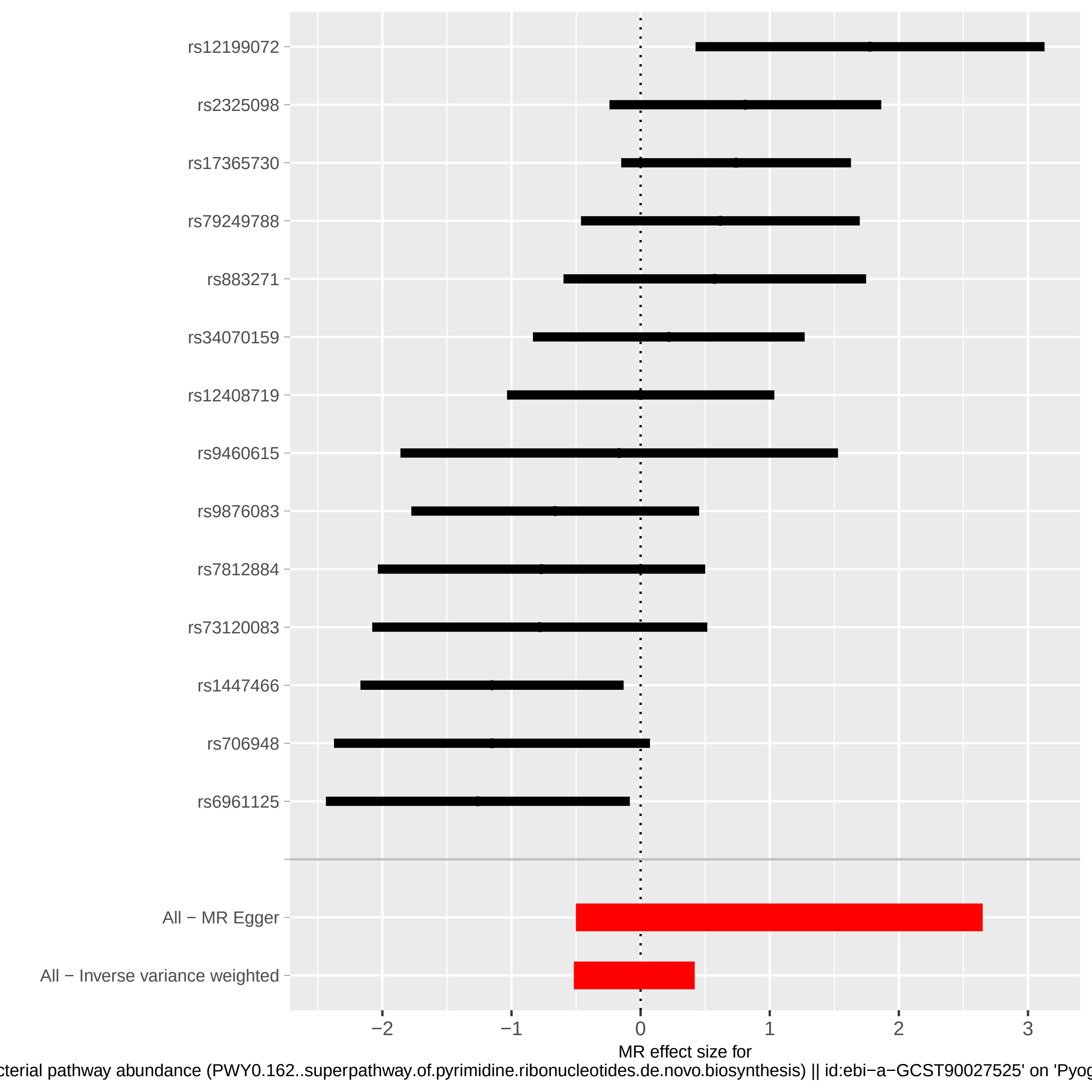
rs12199072
rs2325098
rs17365730
rs79249788
rs883271
rs34070159
rs12408719
rs9460615
rs9876083
rs7812884
rs73120083
rs1447466
rs706948
rs6961125
All − MR Egger
All − Inverse variance weighted
−2
−1
0
1
2
3
MR effect size for
cterial pathway abundance (PWY0.162..superpathway.of.pyrimidine.ribonucleotides.de.novo.biosynthesis) || id:ebi−a−GCST90027525' on 'Pyog
